# Development of fermented rice cake containing strawberry showing anti-inflammatory effect on LPS-stimulated macrophages and paw edema induced mice

**DOI:** 10.1371/journal.pone.0276020

**Published:** 2022-10-13

**Authors:** Chaiwat Monmai, JeongUn Choi, Weerawan Rod-in, Tae Ho Lee, Woo Jung Park

**Affiliations:** 1 Department of Marine Food Science and Technology, Gangneung-Wonju National University, Gangneung, Gangwon, Korea; 2 Department of Wellness-Bio Industry, Gangneung-Wonju National University, Gangneung, Gangwon, Korea; 3 Department of Power Plant, Korea Polytechnic College (Mokpo Campus), Muan-gun, Jeollanam-do, Korea; Universidade Federal do Rio de Janeiro, BRAZIL

## Abstract

Strawberry (*Fragaria ananassa*) is one of the richest sources containing a wide variety of nutritive compounds. Anti-inflammatory activities of fermented rice cake made of strawberry powder as well as rice powder were evaluated. The fermented rice cake containing strawberry powder (SRC) significantly and dose-dependently inhibited NO production in LPS-stimulated RAW264.7 cells without cytotoxicity. Also, SRC effectively suppressed inflammatory gene expression, including *iNOS*, *COX-2*, *IL-1β*, *IL-6*, and *TNF-α*. In addition, the production of PGE_2_, IL-1β, IL-6, and TNF-α was significantly reduced. Furthermore, the anti-inflammatory effect of SRC was investigated using carrageenan-induced paw edema of ICR mice. It was demonstrated that pre-orally administration of SRC at a dose of 50 and 100 mg/kg BW significantly inhibited paw edema induced by carrageenan. This study suggested that the anti-inflammation activities of strawberry rice cake give the potential for increasing the commercialization of rice cake and rice products.

## Introduction

Rice cakes are the popular traditional foods in Asian countries such as Korea and China [1]. They are mainly made with rice flour or other grains and they are prepared by grinding, streaming, boiling, or frying from the different ingredients and different manufacturing process [2–4]. Many studies have developed functional products by adding the powder of several ingredients into rice cakes such as mulberry, sweet potato, and ginseng [5–7]. Some powders such as almond, maqui berry, pumpkin, and chickpea [8–11], have been reported to exhibit antioxidant effects, while the anti-inflammatory effects in fermented rice cake by adding some ingredients have not been extensively studied.

Inflammation is a complex mechanism of interactions among soluble immune factors and related cells that can occur in any tissue in response to traumatic, infectious, post-ischemic, toxic, or autoimmune [12]. It is a protective biological response to harmful stimulation, pathogens, or irritants in vascular tissues that attempts to eliminate infectious stimulation [13]. Macrophages are considered to play essential roles in inflammation [14], and interleukin-1β (IL-1β), IL-6, and tumor necrosis factor-α (TNF-α) are produced by activated macrophages, which are the important mediators of the inflammatory response, and are involved in a variety of cellular activities, including cell proliferation, differentiation, and apoptosis [15, 16].

Strawberry (*Fragaria ananassa*) contains the bioactive compounds of phenolic compounds, vitamins, minerals, polysaccharides, that there have many biological activities [17]. Lin and Tang reported that strawberries showed immunomodulatory activity by stimulated splenocyte proliferation from BALB/c mice [18]. In addition, strawberry juice exhibited the anti-inflammatory effect on murine peritoneal macrophage [19] and also inhibited ROS and NO production in LPS-stimulated RAW264.7 macrophage cells by strawberry extracts [20]. The preliminary research study showed that a mixture of rice powder and strawberry powder containing a ratio of 10: 90 effectively inhibits inflammatory responses [21]. In the present study, the anti-inflammatory properties of a rice cake made from a mixture of strawberry and rice powder (10: 90) were investigated *in vitro* macrophage and *in vivo* mouse models.

## Materials and methods

### Fermented strawberry rice cake sample preparation

Fermented strawberry rice cake (SRC), L-glutamine rice cake (LRC), and rice cake (RC) were provided from Sanghwa F&B Inc. (Gangneung, Korea) after manufacturing. Briefly, SRC and LRC were made from the mixture of rice cake (90%) and strawberry powder or L-glutamine (10%) as described in our previous report [21]. SRC, LRC, and RC were refrigerated for 24 h and then they were lyophilized using a freeze dryer. The freeze-dried rice cake was ground in a blender and was collected as power for further experiments. All samples (SRC, LRC, RC, strawberry powder, and L-glutamine) were dissolved with deionized sterile before use.

### Animal cell culture and sample treatments

Rodent macrophages, RAW264.7 cells were obtained from Koran Cell Line Bank (KCLB, Cat# 40071, RRID: CVCL_0493). The cells were maintained at 37°C in a humidified incubation with 5% CO_2_ in RPMI (Gibco^TM^, Waltham, USA, Cat# 11875–093) supplemented with 10% fetal bovine serum (FBS, Welgene, Korea, Cat# S001-07) and 1% streptomycin/ampicillin (Welgene, Korea, Cat# LS202-02). RAW264.7 cells (1×10^5^ cells/ well) were treated with the various concentrations of SRC or LRC (0.78, 1.56, 3.12, and 6.25 mg/mL), with the control; strawberry powder (STP; 0.625 mg/mL), RC (5.625 mg/mL), and L-glutamine (Gln; 0.625 mg/mL) for 1 h. After that, the cells were stimulated with 1 μg/mL of lipopolysaccharides (LPS from *Escherichia coli* O111:B4, Sigma-Aldrich, USA, Cat# L4391-1MG) and incubated for another 24 h.

### Measurement of cell proliferation and NO production

After incubation 24 h, the cultured medium and Griess reagent (Promega, WI, USA, Cat# G2930) was used for the evaluation of NO production [22]. The cell proliferation was analyzed using EZ-Cytox Cell Viability Assay Kit Kit (DaeilLab Service, Seoul, Korea, Cat# EZ-3000) as described by Kim et al. [23].

The cellular proliferation ratio (%) was calculated based on the following formula:

$$M\mathrm{acrophage}\;\mathrm{proliferation}\;\mathrm{ratio}\;(\%)=\frac{\mathrm{the}\;\mathrm{absorbance}\;\mathrm{of}\;\mathrm{the}\;\mathrm{test}\;\mathrm{group}}{\mathrm{the}\;\mathrm{absorbance}\;\mathrm{of}\;\mathrm{the}\;\mathrm{control}\;\mathrm{group}}x\;100$$

### Analysis of mRNA expression by quantitative real-time PCR

The cells were extracted from the total RNA using Tri reagent^®^ (Molecular Research Center, Cincinnati, OH, USA, Cat# TR118). The total RNA was synthesized to the first-strand cDNA by the High-capacity cDNA reverse transcription kit (Applied Biosystems, Foster City, CA, USA, Cat# 4368814), according to the manufacturer’s instructions. Real-time PCR was subsequently performed using the QuantStudio™ 3 FlexReal Time PCR System (Applied Biosystems, Foster City, CA, USA) and TB Green® Premix Ex Taq™ II (Takara Bio Inc., Shiga, Japan, Cat# RR820A). These reactions were conducted using the primers specific to the target gene of *iNOS*, *COX-2*, *IL-1β*, *IL-6*, *TNF-α*, and *β-actin*, that the sequences were summarized in Table 1.

**Table 1:** Oligonucleotide primers used to assess anti-inflammatory effect of fermented rice cake.

Gene	Accession No.	Primer Sequence (5’ to 3’)	
		Forward primer	Reverse primer
iNOS	BC062378.1	TTCCAGAATCCCTGGACAAG	TGGTCAAACTCTTGGGGTTC
IL-1β	NM_008361.4	GGGCCTCAAAGGAAAGAATC	TACCAGTTGGGGAACTCTGC
IL-6	NM_031168.2	AGTTGCCTTCTTGGGACTGA	CAGAATTGCCATTGCACAAC
COX-2	NM_011198.4	AGAAGGAAATGGCTGCAGAA	GCTCGGCTTCCAGTATTGAG
TNF-α	D84199.2	ATGAGCACAGAAAGCATGATC	TACAGGCTTGTCACTCGAATT
β-actin	NM_007393.5	CCACAGCTGAGAGGAAATC	AAGGAAGGCTGGAAAAGAGC

### Measurement of PGE_2_, TNF-α, IL-1β and IL-6

After treatment, the supernatants were collected and centrifuged at 1000 x g for 20 min. In accordance with the manufacturer’s instructions, the concentrations of PGE_2_, IL-1β, IL-6, and TNF-α were determined by the PGE_2_ ELISA kit (Enzo Life Sciences, Inc. USA, Cat# ADI-900-001), IL-1β (Abcam, USA, Cat# ab197742), IL-6 (Abcam, USA, Cat# ab100712), and TNFα (Abcam, USA, Cat# ab208348), respectively.

### Animals

Male ICR mice with 28 ± 2 g of body weight (BW) were purchased from Orient Bio Inc. (Seongnam, Korea). The animals were kept under controlled conditions with a standard laboratory diet and water for one week before starting the experiment. These experimental protocols were approved by the Institutional Animal Care and Use Committee (IACUC) of Gangneung-Wonju National University, Korea (Approval Number: GWNU-2018-20).

### Carrageenan-induced paw edema in ICR mice

To determine the anti-inflammatory effects in an animal model, carrageenan-induced paw edema was investigated [24, 25]. The animals were randomly divided into six groups (5 mice for each group). Group A received saline solution as control, Group B received STP at 10 mg/kg BW, Group C received RC at 100 mg/kg BW, Group D received LRC at 100 mg/kg BW, Group E-F received SRC at the dose of 50 and 100 mg/kg BW. All treatments were administered orally in mice. After 1 h of oral administration, the suspension of carrageenan at 0.5 mg/25 μL (Sigma-Aldrich, USA, Cat# C1867-1G) was injected into the subplantar tissue of the right hind paw, while the left hind paw was injected with saline solution. Paw edema was measured after carrageenan injection at 90, 180, 270, and 360 min to assess the difference in footpad thickness between the right and left foot.

### Statistical analysis

Data were analyzed by One-way ANOVA (Holm–Sidak post hoc multiple comparison test) under the software of ‘Statistix 8.1’ Statistics (Tallahassee, FL, USA). The statistical differences were considered significant at *p < 0*.*05*.

## Results

### Effects of SRC and LRC on LPS-induced cell cytotoxicity and NO production in RAW264.7 cells

**Fig 1:**
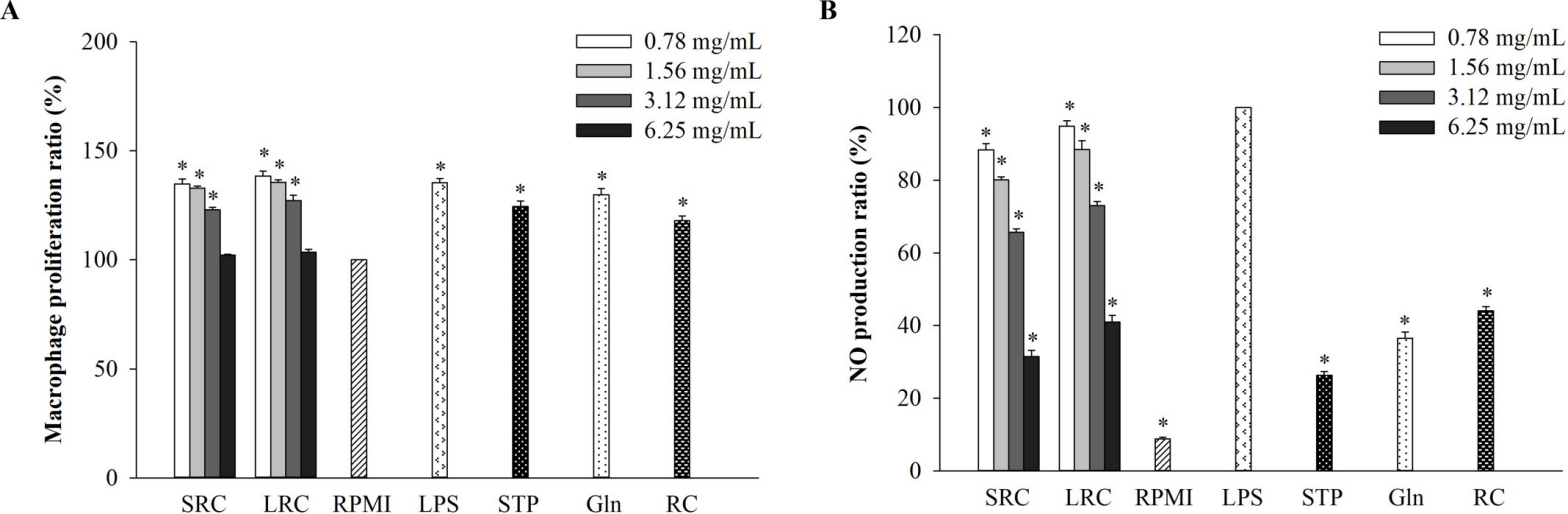
Effect of SRC and LRC on LPS-stimulated RAW264.7 macrophage cells. (A) Macrophage proliferation. (B) NO production. The results are presented as the mean ± SD (*n* = 3). Significant differences are *p* < 0.05 compared with RPMI. SRC = strawberry rice cake, LRC = L-glutamine rice cake, STP = strawberry powder, L-glutamine = Gln and RC = rice cake.

The cytotoxicity of SRC and LRC was determined using RAW264.7 cells with the treatment of different concentrations of rice cake samples as well as negative and positive controls. Cellular proliferation of SRC and LRC treated cells was shown in Fig 1A, in which any samples did not provide any toxicity to RAW264.7 cells.

In addition, the anti-inflammatory effects of SRC and LRC were analyzed using the production of NO production as an important factor for inflammation [26]. Fig 1B shows that LPS led to inflammation by stimulating NO production compared to the normal RAW264.7 cells. The treatment of SRC and LRC significantly inhibited LPS-induced NO production according to the SRC and LRC concentration. Moreover, the treatment with 6.25 mg/mL of SRC and LRC gave lower NO production compared to the normal rice cake group.

### Effects of SRC and LRC on LPS-induced mRNA expressions of immune-associated genes in RAW264.7 cells

**Fig 2:**
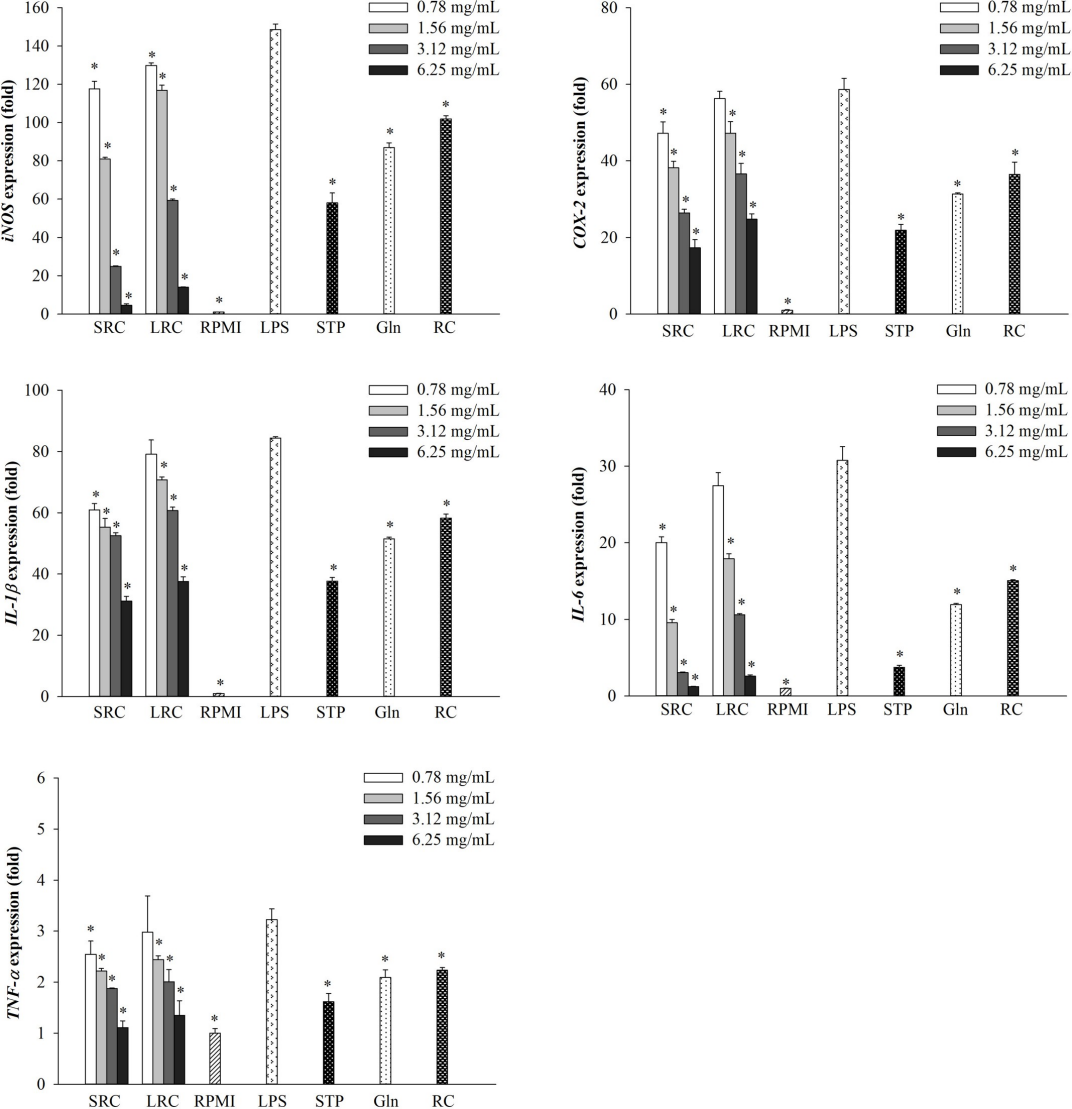
Effect of SRC and LRC on the mRNA expression of *iNOS*, *COX-2*, *IL-1β*, *IL-6*, and *TNF-α* in LPS-stimulated RAW264.7 cells. The results are presented as the mean ± SD (*n* = 3). Significant differences are *p* < 0.05 compared with LPS. SRC = strawberry rice cake, LRC = L-glutamine rice cake, STP = strawberry powder, L-glutamine = Gln, and RC = rice cake.

As shown in Fig 2, *iNOS* and *COX-2* expressions, critical inflammation-associated genes were concentration-dependently inhibited in LPS-induced and SRC and LRC treated macrophage cells. Similar to the mRNA expression of *IL-1β*, *IL-6*, and *TNF-α* which are the pro-inflammatory cytokines [14], were also dose-dependently decreased (Fig 2). In addition, both SRC and LRC treatments at 6.25 mg/mL exhibited the highest anti-inflammatory effect, when compared with the strawberry powder, L-glutamine, and rice cake treatments.

### Effects of SRC and LRC on LPS-induced production of PGE_2_, IL-1β, IL-6, and TNF-α

**Fig 3:**
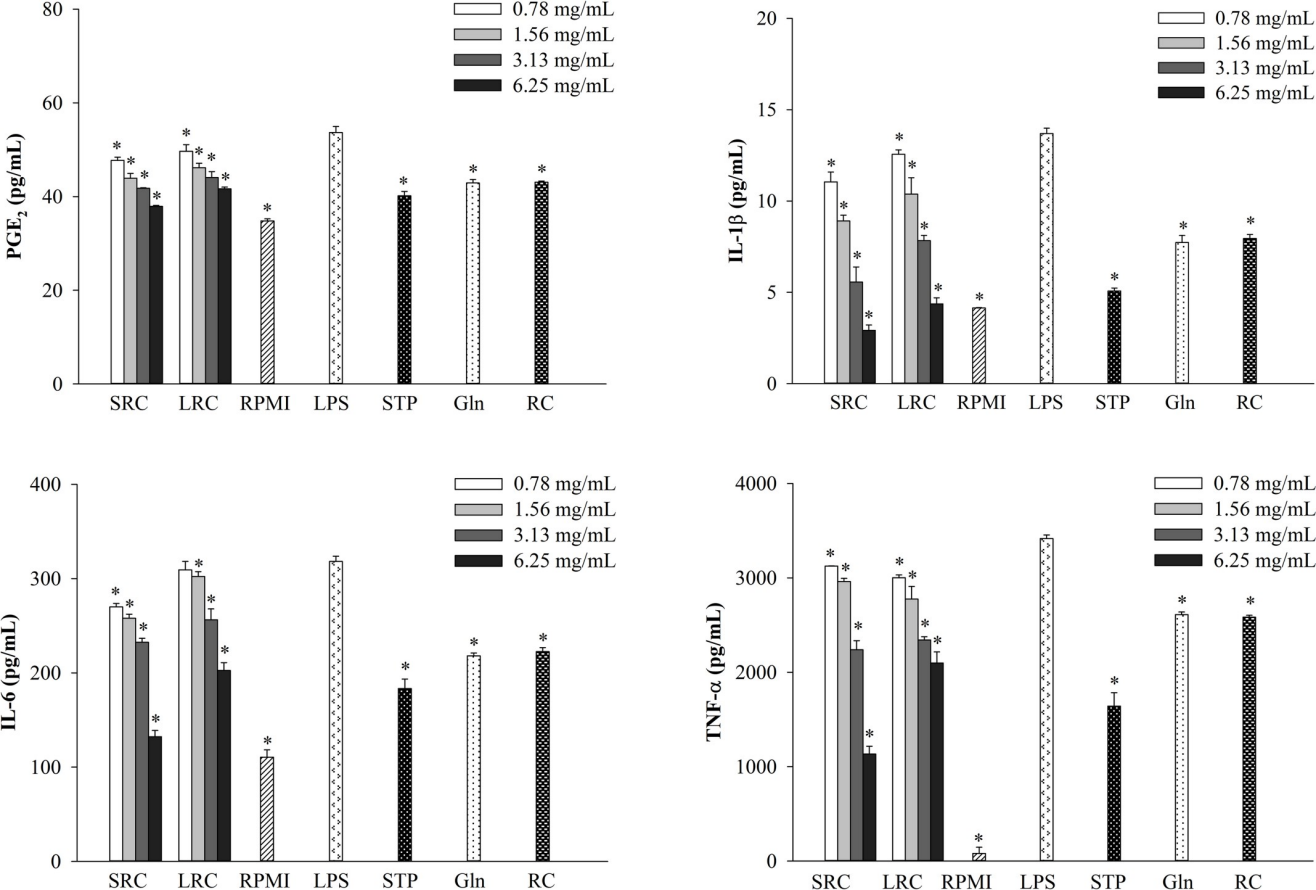
Effect of SRC and LRC on the production of PGE_2_, IL-1β, IL-6, and TNF-α in LPS-stimulated RAW264.7 cells. The results are presented as the mean ± SD (*n* = 3). Significant differences are *p* < 0.05 compared with LPS. SRC = strawberry rice cake, LRC = L-glutamine rice cake, STP = strawberry powder, L-glutamine = Gln, and RC = rice cake.

In order to evaluate the anti-inflammatory effects of SRC and LRC on LPS-stimulated RAW264.7 cells, the concentrations of pro-inflammatory cytokines and PGE_2_ were measured using an ELISA. As shown in Fig 3, the levels of PGE_2_, IL-1β, IL-6, and TNF-α were significantly increased by the LPS treatment. Furthermore, treatment with both SRC and LRC reduced levels of all four in a dose-dependent manner.

## Effect of SRC on carrageenan-induced paw edema

**Fig 4:**
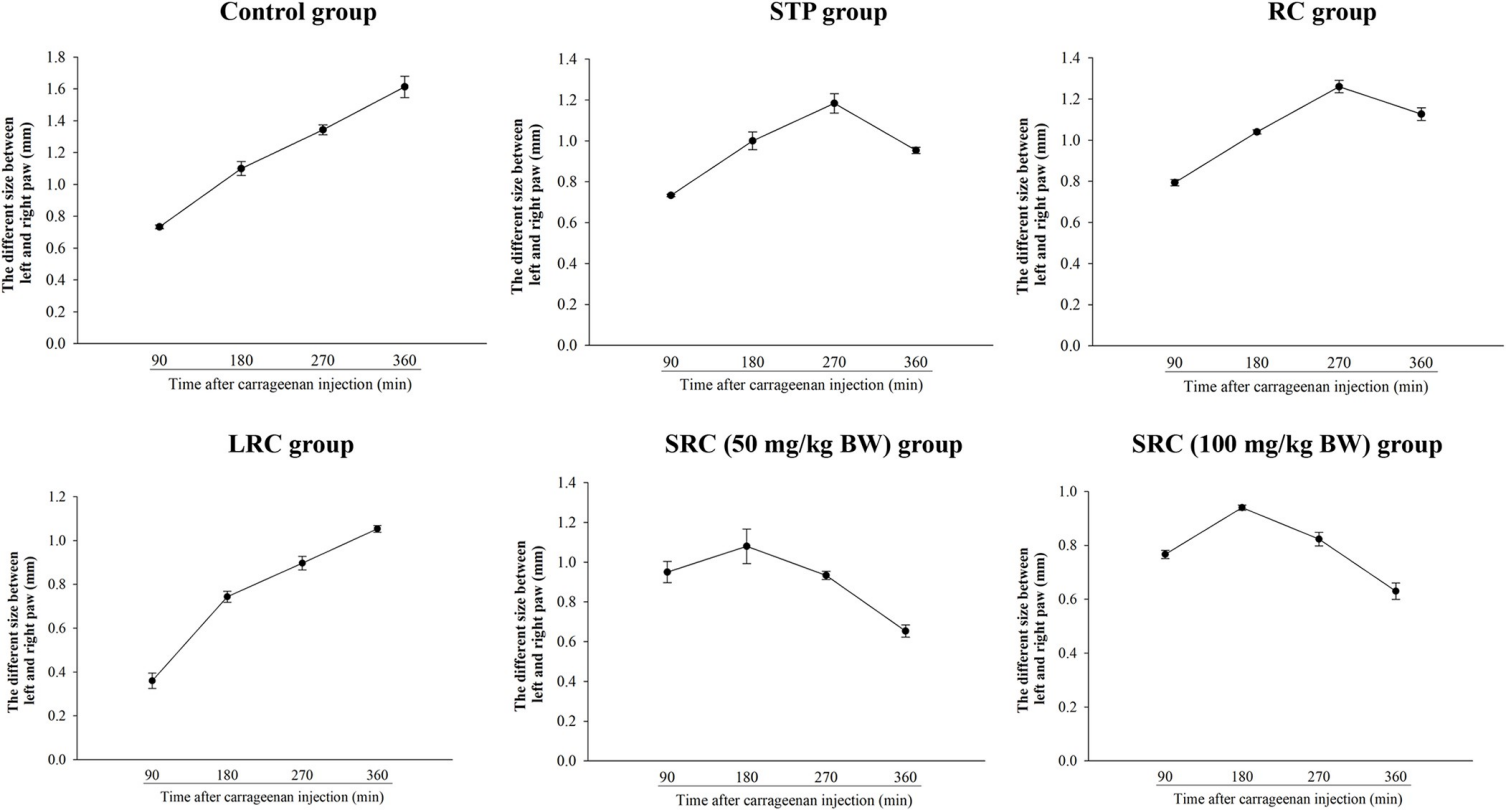
Effect of SRC on carrageenan-induced paw edema. The results are presented as the mean ± SD (*n* = 5). STP = strawberry powder, RC = rice cake, LRC = L-glutamine rice cake, and SRC = strawberry rice cake.

The present study was carried out to evaluate the anti-inflammatory effect of SRC in a model of inflammation using the carrageenan-induced inflammation mice system. Fig 4 and Table 2 showed that oral administration of SRC at the dose of 50 and 100 mg/kg BW reduced the size of paw edema induced by carrageenan. After carrageenan injection, the size difference between the left and right paw was gradually increased over a period of 6 h in the control group (received saline). In contrast to SRC administration, the difference between left and right paw reached the maximum at 2 h after the induction of carrageenan, and then gradually decreased. Moreover, the administration of strawberry and rice cake reduced the difference between left and right paw after 4.5 h of carrageenan induction.

**Table 2:** The different size between left and right paw (mm).

Group	Treatment	Dose	Time after carrageenan-induced (min)			
		(mg/kg BW)	90	180	270	360
A	Control	-	0.73±0.012	1.10±0.044	1.34±0.031	1.61±0.067
B	STP	10	0.73±0.006	1.00±0.044	1.18±0.047*	0.95±0.015*
C	RC	100	0.79±0.015	1.04±0.010	1.26±0.030	1.13±0.031*
D	LRC	100	0.36±0.035*	0.74±0.025*	0.90±0.031*	1.05±0.015*
E	SRC	50	0.95±0.053*	1.08±0.087	0.93±0.021*	0.65±0.031*
F	SRC	100	0.77±0.015	0.94±0.010*	0.82±0.025*	0.63±0.030*

* *p* < 0.05 when compared with control group. Results represent the mean ± SD of 5 animals for each group (*n* = 5).

## Discussion

Inflammation is a defense and prevention against harmful stimuli. The immune response to bacterial infections is induced by LPS by triggering a variety of intracellular signaling events [27]. Macrophages are mainly involved in acute and chronic inflammatory responses which produce NO to enhance to eliminate microorganisms or to regulate inflammation [28, 29], however, excessive production of NO is considered toxic to the host tissue [30]. Various compounds from plants have been reported to play potential roles in many pharmacological properties including anti-inflammatory, anti-cancer, antioxidant, cardiovascular, and neurological diseases [31–33].

Strawberry is reported to have an anti-inflammatory effect via reducing the production of NO and inhibiting the inflammatory mediators and cytokines in LPS-stimulated RAW264.7 cells [34–37]. Strawberry extract exhibited anti-inflammatory effects, reducing the NO and ROS intracellular production as well as inflammatory markers (TNF-α, IL-1β, IL-6 and, IL-10) through the activation of the Nrf2 pathway, and NF-κB signaling pathway in *in vitro* LPS stimulation [20]. Strawberry also significantly reduced the inflammation-associated biomarkers in the clinical study [32, 38]. Similarly, serum collected from rodents fed blueberry and strawberry-enriched diets showed anti-inflammatory activity [39]. Previous research examined the anti-inflammatory activities of a strawberry-rice powder mixture as a material of fermented rice cake on RAW264.7 cells induced by LPS and mouse models induced by carrageenan [21]. Nevertheless, no research has been reported on the anti-inflammatory effects of rice cake supplemented with strawberry powders, so our experiments used LPS-stimulated RAW264.7 cells and carrageenan-induced inflammation mice to investigate the anti-inflammatory properties of rice cake supplemented with strawberry powders.

The current study showed that the supplementation with strawberry powders in fermented rice cake exhibited anti-inflammatory activity. NO production, critical immune-regulatory biomarker for inflammation [40], was significantly decreased according to SRC concentration (Fig 1B). The pro-inflammatory cytokines such as IL-1β, IL-6, and TNF-α have been known to regulate immune systems in macrophages [15, 16]. The current study also showed that the expression of *IL-1β*, *IL-6*, and *TNF-α* was significantly and dose-dependently decreased in LPS-stimulated RAW264.7 cells when the cells were pre-treated with SRC (Fig 2). Among the fermented products, red mold rice by *Monascus purpureus*, was reported to inhibit NO production in LPS-stimulated RAW264.7 cells [41], and the pelargonidin-3-O-glucoside, ellagic acid, and polyphenolic extracts, which were isolated from strawberry also inhibited the production of NO, TNF-α, and IL-6 and the expression of pro-inflammatory cytokines [34–37]. Moreover, our results showed COX-2 which is a key mediator of inflammatory pathway [42] also significantly inhibited in LPS-stimulated RAW264.7 cells. Similarly, the LPS-induced *COX-2* expression was decreased by the polyphenolic extracts from strawberry [37].

Injection of carrageenan into the mouse paw leads to local inflammation which is a suitable method for evaluating anti-inflammatory agents [43]. Carrageenan-induced rat paw edema is a widely used test to determine the anti-inflammatory activity [44–46]. Mouse paw edema has been increasingly used to test new anti-inflammatory drugs as well as to study the mechanisms involved in inflammation [45]. Our results showed that the anti-inflammatory effect was observed after carrageenan-induced inflammation in mice that received SRC via reducing the size difference between the left and right paw of SRC-treated mice. These results are similar to some reports which showed the anti-inflammatory activity of Berberidacceae roots extracts as well as *Berberis* root and bark extract on carrageenan-induced mice paw [25, 47–49]. However, any reports which studied fermented rice cake containing anti-inflammatory effects have not been found.

## Conclusions

Our results demonstrated that SRC exerts anti-inflammatory effects both *in vitro* and *in vivo* physiological systems. SRC inhibited the LPS-induced NO production and pro-inflammatory cytokines on RAW264.7 macrophages cells. Furthermore, SRC also suppressed the paw edema thickness on the carrageenan-induced mice model. Therefore, these results suggested that fermented rice cake using strawberry powder has a potential traditional food to provide anti-inflammatory effects under several disease conditions as a supplementary diet.
